# The effect of suppressing funeral rituals during the COVID-19 pandemic on bereaved families^*^

**DOI:** 10.1590/1518-8345.4519.3361

**Published:** 2020-09-07

**Authors:** Érika Arantes de Oliveira Cardoso, Breno César de Almeida da Silva, Jorge Henrique dos Santos, Lucas dos Santos Lotério, Aline Guerrieri Accoroni, Manoel Antônio dos Santos

**Affiliations:** 1 Universidade de São Paulo, Faculdade de Filosofia, Ciências e Letras de Ribeirão Preto, Ribeirão Preto, SP, Brazil.

**Keywords:** Coronavirus Infections, SARS Virus, Pandemics, Bereavement, Mental Health, Funeral Rites, Infecciones por Coronavirus, Virus del SRAS, Pandemias, Aflicción, Salud Mental, Ritos Fúnebres, Infecções por Coronavírus, Vírus da SARS, Pandemias, Luto, Saúde Mental, Rituais Fúnebres

## Abstract

**Objective:**

amidst the greatest health crisis in history triggered by COVID-19, this documental study was intended to understand the meanings individuals who have lost loved ones in this context assign to the phenomenon of suppressed funeral rituals.

**Method:**

based on the theory of grief, the *corpus* of this study was composed of documents published in digital media containing personal writings and reports of experiences freely and easily available to the public. Two researchers with expertise in the field used inductive thematic analysis to interpret data.

**Results:**

the experiences shared in the reports reflect the suffering experienced by the sudden death of a significant person, which is amplified by the absence or impediment to performing familial farewell rituals. The suppression or abbreviation of funeral rituals is a traumatic experience because family members are prevented from fulfilling their last homage to the loved one who has suddenly passed away, causing feelings of disbelief and indignation.

**Conclusion:**

alternatives and new ways to celebrate passage rituals in emergencies of strong social commotion such as a pandemic are needed to provide support and comfort to family members, friends, and relatives. These rituals help survivors to overcome the critical moment, decreasing the risk of developing complicated grief.

## Abstract (es)

**Objetivo:**

en medio a la mayor crisis sanitaria de la historia, deflagrada por la pandemia de COVID-19, este estudio documental tuvo por objetivo comprender los sentidos atribuidos al fenómeno de la supresión de rituales fúnebres por personas que sufrieron pérdidas de seres queridos en ese contexto.

**Método:**

amparado en la teoría del luto, fue constituido el *corpus* de investigación a partir de documentos publicados en los medios de comunicación digital, conteniendo escritos personales y relatos de experiencias abiertas al público. El análisis temático inductivo de los textos fue realizado por dos investigadores con pericia.

**Resultados:**

la experiencia compartida en las declaraciones repercute el padecimiento por la muerte repentina de persona significativa, ampliado por la ausencia o truncamiento de rituales familiares de despedida después de la muerte. La supresión o abreviación de rituales fúnebres es vivida como una experiencia traumática, ya que los familiares se encuentran impedidos de cumplir las últimas homenajes al ser que se fue súbitamente, generando sentimientos de incredulidad e indignación.

**Conclusión:**

es necesario crear alternativas y reinventar maneras de celebrar los rituales de pasaje en situaciones de emergencia de fuerte conmoción social como una pandemia, de modo a ofrecer amparo y confort a los familiares, amigos y parientes. Esto auxilia a los supervivientes a superar el momento crítico, reduciendo el riesgo de desarrollar un luto complicado.

## Abstract (pt)

**Objetivo:**

em meio à maior crise sanitária da história, deflagrada pela pandemia de COVID-19, o objetivo deste estudo documental foi compreender os sentidos atribuídos ao fenômeno da supressão de rituais fúnebres por pessoas que amargaram perdas de entes queridos, nesse contexto.

**Método:**

com amparo da teoria do luto, foi constituído o *corpus* de pesquisa a partir de documentos publicados na mídia digital, contendo escritos pessoais e relatos de experiências abertos ao público. A análise temática indutiva dos textos foi realizada por dois pesquisadores com *expertise*.

**Resultados:**

a experiência compartilhada nos depoimentos repercute o padecimento pela morte repentina de pessoa significativa, ampliado pela ausência ou truncamento de rituais familiares de despedida após o óbito. A supressão ou abreviação de rituais fúnebres é vivida como uma experiência traumática, pois familiares se veem impedidos de cumprirem suas últimas homenagens ao ente que se foi subitamente, gerando sentimentos de incredulidade e indignação.

**Conclusão:**

é preciso criar alternativas e reinventar maneiras de celebrar os rituais de passagem em situações emergenciais de forte comoção social como uma pandemia, de modo a oferecer amparo e conforto aos familiares, amigos e parentes. Isso auxilia os sobreviventes a superarem o momento crítico, reduzindo o risco de desenvolvimento do luto complicado.

## Introduction

The World Health Organization (WHO) declared on March 11^th^, 2020 that the COVID-19 outbreak, caused by the novel coronavirus (SARS-CoV-2), characterized a pandemic situation^(1-2)^. It is estimated that 80% of the infected individuals develop mild or moderate forms of the infection while 20% manifest the severe version of the disease. Of these, 5% manifest the most severe form, which can rapidly progress to severe acute respiratory syndrome and other complications that may lead to death^(3)^.

There is a great concern worldwide with the novel coronavirus’ high rate of transmissibility, which has caused different and devastating impact^(4-5)^, forcing local governments to establish, in addition to health emergencies and states of calamity, exceptional administrative measures for funeral services. As a result of the crisis, traditional rituals to honor the dead and comfort mourners needed to be abbreviated or even interrupted. Therefore, the COVID-19 pandemic has demanded many aspects of the dying experience and its rituals to be reformulated in the Eastern and Western worlds^(6)^.

Psychology has long recognized the emotional value and structuring role of rites and rituals in different societies and cultures. Rites comprise a broader category, including rites of passage or healing, while a ritual is a set of gestures and actions that make up the rites. Human rituals are common to all peoples and are symbolic actions, repetitive, standardized, and highly valued behaviors that help individuals to channel emotions, and share beliefs and transmit values^(7-8)^. Marking the transience of life, funeral rituals have always been present in history^(9-11)^ to demarcate a state of mourning, acknowledging the value and importance of those who have passed away, favoring change of roles and allowing the transition of the cycle of life^(10)^. One should also consider the role funeral rituals play in psychological maturation, as they help individuals to face concrete loss and trigger a grieving process, allowing people to publicly manifest their grief^(10)^.

A lack of rituals when a physical body parts makes it difficult to psychologically acknowledge the loss. Additionally, sudden and unexpected deaths prevents mourners to prepare themselves to deal with the loss, considering that physical death does not accompany social and psychological death, which may lead people to face difficulties when facing their mourning process. When intense, these barriers may favor the so-called *complicated grief*, characterized by long-lasting disorganization that makes it difficult or impede psychological reorganization and the resumption of life. There may be also exacerbated symptomatic manifestations, such as an expression of intense feelings, somatization, social isolation, depressive episodes, low self-esteem, self-destructive impulse^(12)^, frequent thoughts directed to the deceased, inability to accept loss, self-blame, and difficulty imagining a meaningful future without the loved one^(13)^.

The elements that contribute to complicated grief may be divided into factors of personal risk, associated with the history and prior events in the life of the bereaved individual, and risk factors related to the circumstances of the death of the loved one. The latter comprise the death of a child or youth, death of wife or husband, lack of psychological preparedness to deal with death, death in a hospital, among others^(14-16)^. On the other hand, protection factors against complicated grief include the availability of psychological and social support^(14)^, clear communication between the health staff and family members of the deceased^(17)^, demonstration of empathy by other family members and the community^(18)^, and the meanings assigned to the death of the loved one^(19-21)^.

There are many factors in the context of the COVID-19 pandemic that may hinder one’s grief, such as sudden death, being totally isolated in a hospital facility, the experience of death in a situation of intense distress and physical pain, less time available for people to give meaning to the loss, exposure to stigma and social discrimination, fewer rites and rituals, lack of social support, family relationships subject to greater tension, and other losses that occur simultaneously with death. In this adverse context, interventions mediated by the use of digital technologies have been proposed to alleviate the suffering of families and friends^(22)^.

This study’s objective was to understand the meanings individuals who experienced the loss of loved ones in the context of the COVID-19 pandemic assigned to the phenomenon of suppressed funeral rituals.

## Method

Documentary research with a qualitative approach. Qualitative studies can adopt different instruments to compose the research *corpus*, among which documents (written material) that have not been previously analyzed or systematized. This type of study is intended to analyze the content of the documents chosen to compose the study *corpus* by establishing a hypothesis of interest, being considered an important resource in qualitative research in which documents are means of communication^(23)^. The type of the document chosen will depend on the study objective and the great challenge of researchers is to know how to select and treat data and interpret the results^(24)^. This type of study is intended to produce new knowledge by exclusively using the document analysis method. Researchers need to understand documents as a means of communication and take into account the fact that documents were developed considering that someone who would have access to their content^(25)^.

In this study, the documents addressed include: written personal documents, freely available to the public, and not produced specifically for this study, that is, the *corpus* was composed of texts that existed even before this study was planned^(23)^. These are spontaneous productions, with the intent to communicate or share a given experience in blogs or virtual social media, or are narratives produced in the context of news aired on digital media. The reports of bereaved families available on the Internet were selected according to the following inclusion criteria:

- reports and testimonies posted on public websites from March 1^st^ to April 20^th^, 2020. This period was chosen to comprise the beginning of the COVID-19 pandemic in Brazil;
- reports and testimonies concerning illness, death of a significant one and a lack of rituals after death;
- reports of close family members and relatives that show an apparent and clear affective bond with the deceased person;

Exclusion criteria were:

- content that did not mention the experience of illness of a family member and the experience after his/her death;
- reports of health workers or workers from other correlated fields;
- reports not shared on digital websites in the national context.
- content with restricted access, that is, that could only be accessed by subscription or friendship request or some other form of authorization

These criteria were established in line with this study’s proposal and theoretical framework (theory of grief). The following question was established: “*What meanings do people who lost family members due to COVID-19 assign to the phenomenon of suppressed funeral rituals?”*

Brazilian websites freely and openly available to the public, which had aired content concerning experiences with the pandemic, were previously selected to delimit this study’s search universe. In addition to being easily accessible, the websites and materials were chosen considering the criterion of impact, which was estimated by the number of accesses, likes, and interactions with the content posted. After selecting the texts of interest in line with the study’s objective, the excerpts were collected that reported discourse, experiences, and testimonies shared by adult individuals concerning how they were dealing with the illness and loss of family members.

The websites used to collect data were the Twitter and Google platforms. Twitter makes available an “advanced search” tool with which one can select the period of the publications, language, degree of interaction, words, and even specific phrases. News on the Google platform was filtered by openly accessible news reporting losses due to complications caused by COVID-19, whether confirmed or suspected. The following combinations of search terms were used in Twitter: “Covid19” and “Mourning”, “Covid19” and “Funeral”, “Covid19” and “Loss”, “Covid19” and “Sad”, and also a combination of “Coronavirus” and “Mourning”, “Coronavirus” and “Funeral”, “Coronavirus” and “Loss”, “Coronavirus” and “Sad”. Publications should be written in Portuguese and were selected if there was at least one like. These publications were freely accessed on the Twitter platform so that any individual could access them without necessarily having to follow the profile in which the publication was posted or to ask authorization to see it.

Two researchers independently conducted the search on April 21^st^, 2020. The reports extracted from the news that appeared in the initial search filter were fully read against the background of the guiding question and selected according to the inclusion/exclusion criteria. Each text was included in a form addressing: title, participants’ characteristics, electronic address of the publication, and testimonies collected. The Kappa^(25)^ index was calculated to verify agreement between the two researchers, and a Kappa coefficient of 0.83 indicated almost perfect agreement between them. At the end of this first selection stage, 84 reports were recovered from Twitter and 17 from Google. Then, the texts selected by the researchers were compared and divergences concerning the inclusion or exclusion of content were resolved by consensus.

The material collected was read once again with a view to refining content that strictly answered the study’s objective. Twenty-three publications remained in the final selection and comprised the study’s *corpus*.

The researchers organized and analyzed the reports extracted from the texts selected according to inductive thematic analysis^(26)^, considered an accessible and flexible approach that is appropriate for qualitative research. Its objective is to identify and analyze patterns in the reports collected to interpret the constitutive aspects of the contents.

Two researchers with expertise in the phenomenon of mourning, who had not participated in the previous stages, conducted the analysis, involving: 1) exploration of the material: inspection and exhaustive reading of each text transcribed in sequence; 2) generation of the initial codes; and 3) search for themes. In step 1, the narratives that compose the study’s *corpus* were fully read. In step 2, the researchers coded data, meaning that the texts were read numerous times and content was associated with key-ideas based on the question “what is it that is written here?” Subsequently, the researchers reviewed all the transcriptions to ensure that nothing was left out of consideration and discussed the codes and initial themes with the rest of the team (step 3).

This last step was crucial to the analysis because, in qualitative research, it is not expected that fully exempt researchers reach the same representations of a given event. Rather, some level of agreement, even if temporary, is expected, understanding that this representation of reality is acceptable. This kind of an agreement that different observers may achieve regarding a given phenomenon legitimates the analysis presented^(27)^. The discussions and suggestions indicated the need to expand or change the analysis, establishing and naming the final themes, rigorously obeying what is recommended by the analysis method adopted.

This study was conducted according to the ethical guidelines established in Brazilian Health Council Resolution 510/2016, and the Institutional Review Board approved the project, registered under CAAE 33584520.0.0000.5407. Given this study’s documental nature, the researchers worked with data that had been previously published, without even interacting with the families depicted. Even though the content accessed was public and freely available online, we opted for omitting the participants’ real names and other characteristics that could reveal their identities.

## Results

Six testimonies were chosen of children of deceased individuals, four of cousins, three of mothers, three of daughters-in-law, two of nephews, two of husbands, one of a wife, one of a brother-in-law, and one of a grandson.

### a)Unexpected, frightening, and invisible: death closes its siege

Family members mentioned that the impossibility of providing support to the family member at the time of the diagnosis, especially when the disease worsened, triggered suffering: *COVID-19 arrived in my family. My father’s brother is in the hospital in a severe condition. The saddest of it all is that the family cannot be with the patient, supporting and providing at least some care. True loneliness* (niece).

In general, the contamination of one family member by COVID-19 is accompanied by surprise and perplexity, which aggravate distress because the fantasy of invulnerability is dismantled. This notion results from an irrational belief that bad things only happen to others. Even though the mass media has widely disseminated information, outlining the pandemic scenario, the fact that the virus is invisible contributes to fuel skepticism in the population: *We think it won’t happen to us until it does* (wife, widow of a 35-year-old man); *Unfortunately, my father didn’t take it seriously, he said it was a media thing. When he decided to travel, I warned him not to go, but he did, even though he was aware of the risks because he didn’t believe in the disease. My father was 100% healthy* (daughter).

Unpredictability and fear of the unknown establish a climate of generalized fear. Two possible threats are feared: loss of a family member and loss of a sense of control over events, triggering experiences of helplessness: *She was healthy, had no diseases, was not in the risk group, but this virus does not care about one’s age. It’s not just the elderly. It is not choosing age* (cousin); *He was healthy. They said he was getting better. He did not have diabetes or hypertension, he was a healthy person; didn’t drink or smoke* (brother-in-law).

The pathogen’s invisibility and dangerousness are two additives that enhance persecutory anxiety. A feeling of constant threat and a sense of loss of control over life are directly associated with the impression that the hidden and feared enemy is everywhere and can break through barriers and invade the supposedly safe haven of home: *I never thought that this virus could bring him down. I’m afraid now! Afraid of going out and bring this virus home and contaminate my family* (daughter).

One of the aspects most frequently mentioned in the reports and which seems to be a barrier impeding people from absorbing the initial impact of the loss is the fact that things happen very suddenly. Unpredictable and chaotic, atypical situations begin to proliferate, bringing along a sense of unreality in the face of an overwhelming experience in times of pandemic: *It’s a loss the family didn’t expect, though he did have chronic diseases. Nobody did anything out of the ordinary, didn’t go to an airport, didn’t travel. He was home and the disease ended up inside home and he died in less than a week* (cousin).

The disease’s sudden onset and the irreversible condition that sets in give no opportunity for patients or families to prepare for the worse. This is an especially cruel and tragic facet of COVID-19, a serious disease of which individuals suffer and die alone, a situation of intense distress. One of the consequences is that experiencing such a situation leads families to reconsider certain misconceptions, such as “it is not that serious”, as shown by the testimony of a woman who had lost her 38-year-old cousin a few days before and was facing the death of her father: *Now, with the loss of my father I consider it my duty as a citizen to explain to the world that this virus is not simple flu. A person can be extremely healthy* [and die] (daughter); *It is so much pain seeing a loved one alone in an ICU bed, isolated, feeling abandoned because a cursed virus reaped his lungs, taking his oxygen and immense joy* (daughter-in-law).

### b)Experiencing losses: there is no time to say goodbye, there is no closure

In addition to having to deal with a traumatizing experience of loss, the high risk of becoming infected with the novel coronavirus prevents the family from having a proper funeral: *I hope you never have to stay home inert, while your relative is being cremated without any relative to say goodbye or pay honor* (daughter-in-law); *His body came in a closed bag, he wasn’t buried in a coffin* (mother).

These rites of passage that make up farewell rituals are so naturalized in daily life in the Brazilian culture that their suspension, even if justified, is surrounded by disbelief and distress. The dominating feeling is that a cycle was opened but not closed: *The saddest is that, because we are amidst a pandemic, there will be no funeral in the way he’d love it most, with his orchestra playing* (grandson). The process of mourning the dead, of gathering the family and friends to get comfort and solidarity was summarily interrupted: *It is the saddest thing in the world to lose a child and not be able to go to his burial, not being able to do anything. It is really, really, really difficult. It is unbearable* (mother).

Additionally, with the increase in cases and rapid acceleration of the pandemic, the terrible humanitarian catastrophe is added to a sanitary and funeral emergency: *It was really painful. The funeral home itself was concerned with the procedure. The funeral staff took the coffin to the hospital, put her inside the way she* [was] *in the hospital, sealed it, and took her to* [city’s name]. *The whole thing lasted less than 20 minutes. The grave was already open, waiting* (cousin).

The need of post-death care intended to prevent contagion by the virus figures as another “dehumanization” factor that mischaracterizes the funeral ritual, with burial personnel wearing Personal Protective Equipment (PPEs), shortened funerals, decreased number of people attending the event, and sealed coffin: *Everyone there was wearing masks, the gravediggers were wearing PPEs, it even seemed Chernobyl. We had only 20 minutes* (niece); *My mom’s cousin passed away and there will be no funeral. They will only bury her in a sealed coffin. So much suffering. When will this be over?* (cousin).

Amidst the pandemic, the scenario that is presented gains dramatic contours that potentiate the pain and distress of family members, as it becomes impossible to receive social support due to the stigma associated with the disease: *Now that the isolation period the physician recommended is over, I confess that I’m afraid to go out on the streets. It is as if we may contract a disease that will never be cured* (daughter).

The mourner ceases to be an object perceived as vulnerable, someone who needs support and protection and becomes stigmatized as a potential vector of transmission, a threatening and persecutory object, which further amplifies feelings of loneliness and discouragement: *On top of everything we have to deal with prejudice. An acquaintance, who is aware of all the suffering we are facing, walked to the other side of the sidewalk, afraid of becoming infected. We’ve already heard our neighbors saying that our house was full of “people infected with the coronavirus”* (daughter).

### c)The memory of the last hug: strategies to minimize suffering

In general, the reports are permeated by pain, revolt, perplexity, and feelings of abandonment, but also show the possibility of developing protective actions and mechanisms able to minimize suffering. The families reinforce the importance of establishing a bond of trust with the health staff, especially regarding the sharing of information: *I talked with the physician for 20 minutes, yesterday I spent 45 minutes on the phone. I did not want to hang up because there’s always something missing, one more question to ask. I really wish I could see my wife* (husband).

In addition to communication with the staff, the excerpts show the importance of exercising empathy to deal with the situation: *Reflect, rethink, put yourself in the shoes of many families who are experiencing what our family is now experiencing. Respect people’s pain* (daughter-in-law).

Lack of empathy is reported as one of the greatest difficulties families need to overcome: *There are people infected with the coronavirus who will remain in isolation for 15 days and will then be cured. Unfortunately, there are many people infected with a lack of compassion and love for others. For these, there is no cure* (woman who lost her mother and uncle with COVID-19).

Some families seek comfort in the notion that there may be a greater purpose in the loss of their loved one: *I don’t accept the idea of losing my son due to such a severe problem. I only hope that people believe that this problem exists and is here* (mother); *I think it is important that the death of my wife helps people to take greater care. It is no joke what is happening* (husband).

## Discussion

Various factors have hindered the process of mourning the loss of loved ones among families in the global scenario of the first wave of COVID-19^(28)^: the fact that there is little scientific knowledge about the dynamics of this disease; its high lethality rate^(29)^; the vertiginous speed at which SARS-CoV-2 is spread; in addition to the mandatory restriction of physical proximity, suppressing the ideal conditions for families to receive social support, fomenting the stigma related to the disease. These factors are enhanced by the devastating impacts of the infection on social and economic spheres.

The threat of the pandemic affects each individual in a particular way, according to one’s prior experiences, individual functioning aspects, contexts of life, protective resources, and perceived vulnerability. A movement commonly observed, especially among younger individuals, is that they deny the possibility of possible infection by the novel coronavirus. The use of a defensive resource to minimize the severity of the health emergency and the feeling of being “immune” to COVID-19 can be seen as a way to bear the anguish triggered by a pandemic that threatens everyone indistinctively. When someone close becomes sick, however, the illusion of having omnipotent control over life dissipates and the myth of supposed invulnerability falls apart^(30-31)^.

In addition to dealing with a traumatizing loss, that is, because it happens in the context of acute disease, families are prevented from gradually preparing themselves emotionally, there is a high risk of contamination that impedes individuals to perform a proper funeral. In accordance to the standards established by the *Centro de Vigilância Sanitária* [Health Surveillance Center], the protocol of the *Serviço de Verificação de Óbitos (SVO)* [Coroner’s Office] provides the burial to be quick and watched at a distance by up to 10 individuals, except for children, pregnant women, elderly individuals and those with chronic diseases. The coffin cannot be exposed^(32)^, impeding a ritualization that is a fundamental part of the Brazilian culture.

Therefore, in addition to the brutal loss of a loved one, people experience the impossibility to celebrate the final rites that give the opportunity of communion, complicity, connection with the sacred, and start a necessary process of detachment^(10)^. Paying the last tribute to a loved one is a mental health gesture that allows individuals to make amends and reconcile with life. In the exceptional regime determined by the pandemic, however, funerals were abolished and necessary limitations were imposed on burials, creating disturbances rather than comforting^(10)^. The feeling that remains is that “one stage was skipped”, as it is essential to share grief and receive social support after the death of a loved one^(33)^. These restrictions favor the risk factors for the development of complicated grief or at least maximize difficulties to elaborate normal grief.

Among potential protective factors, families report trust in the health staff, especially regarding the sharing of information. This relationship with workers may be capable to promote the humanization of relationships by providing information, validating messages, and remotely interacting with families^(17)^. Considering the importance of this communication for families, its inclusion in the care process is extremely important. In this sense, researchers have worked on manuals and other didactic resources directed to family members, given the need to plan the flow of communication to mitigate the risks of people developing complicated grief and post-traumatic stress in the context of the pandemic^(22)^.

In addition to the staff establishing communication with families, the excerpts show the importance of establishing bonds and actively exercise empathy to deal with adverse situations. Lack of empathy does not only show through non-recognition and indifference to the pain of the bereaved and which favors disenfranchised grief. It is a highly negative and destructive symptom that erodes the basis of social cohesion and organization, hindering the process of personal, family and collective grief, preventing the construction of a social narrative of loss^(18)^.

In the conflicting scenario of the pandemic, the development of social skills that favor empathic attitudes is essential to deal with the health crisis in the present and future. Campaigns promoting proactive attitudes of solidarity, reinforcing the ability and responsibility of each individual to contribute with their share, show how union in times of collective adversity may make a difference and positively impact the lives of people.

Some family members seek comfort in the belief that the death of the loved one served a greater purpose. This need to assign a noble and humanitarian meaning to the death of a loved one may provide some relief to the experience of an imponderable situation. In fact, in some studies, finding a purpose and meaning for the life that has suddenly ceased is reported to be related to fewer characteristics of complicated grief and lower levels of stress^(19-21)^.

The process of assigning meaning to death, especially in an unexpected and exceptional situation, is seen as a protective factor against complicated grief^(19-21)^. In this sense, it is necessary to encourage care practices and psychosocial support centered on a search for meaning^(14)^, including encouragement to humanitarian campaigns, social solidarity and activism, participation in crisis contingency committees, and volunteer associations to support other bereaved families.

This study presents some limitations. It is important to note that the analysis took into account reports that were available online, which prevents deepening the investigation as it is not possible to dialogue with the people who produced the narratives. This dialogue would enable a deeper analysis beyond what is written so that other layers of meanings assigned to the events, feelings, and experiences could be accessed and interpreted. Future studies are recommended to conduct interviews with those who lost family members, aiming to advance the understanding of what has been produced thus far.

Despite these limitations, this study contributes to improving the practice of professionals working in the context of the pandemic, as it addresses an important topic for the mental health of bereaved family members, who often have their care neglected. The support provided to families and friends, and also to health workers and other workers involved in the tasks linked to funeral rites and rituals, can help mourners to overcome the critical moment, decreasing the risk of developing complicated grief. The greatest danger to be avoided is that the farewell ritual loses its meaning and fails to fulfill its role in aggregating family members in the distillation and psychological elaboration of shared pain.

## Conclusion

Based on the analysis of the reports collected, we could verify that, in addition to serious harm to the mental and physical health of those affected, this novel disease also imposes challenges that need to be considered when planning psychosocial interventions. From the perspective of integral care, actions need to be comprehensive and include both those who became sick and people who, despite remaining healthy or asymptomatic, witnessed family members who faced the more severe form of the disease and even died. After hospitalization, families are prevented from accompanying patients in the hospital, receiving only sparse news over the phone. Families suffer due to being impeded from accompanying their loved ones during their final days in the hospital. After a lonely and helpless death, the suspension of funeral rites and the need to perform a quick burial in the presence of few family members with a sealed coffin empty the farewell rituals. Some stages of the process of establishing meanings are suppressed, which makes it difficult to accept the loss.

Throughout human history, funeral rituals have serve as existential milestones in the process of elaborating and re-signifying the death of a loved one. The acute grieving process triggered by a loss is important for psychological health because it is an opportunity to elaborate on the finite nature – of others and oneself. From the moment in which family members are prevented from performing farewell rituals due to the restrictions imposed by the pandemic, the mourning may become even more painful and even incomplete. It may trigger psychological distress that tends to go on indefinitely, providing raw material for the development of complicated grief.

To mitigate the impact of these problems, which humanity will have to learn to live with from now on in predictable new epidemic outbreaks, it is imperative to innovate how funeral rituals are performed, certainly making them safer to minimize the risk of contamination by the novel coronavirus, and even shortening these events, however, without emptying their meaning. This situation may be remedied with the help of technology, which permits preserving physical distance (which is not necessarily social distance) or even proposing new configurations to celebrate the traditional rituals specific to each culture.
